# Supplementary figures and images for: Haloferax volcanii Immersed Liquid Biofilms Develop Independently of Known Biofilm Machineries and Exhibit Rapid Honeycomb Pattern Formation
Source: mSphere. 2020 Dec 16;5(6):e00976-20. doi: 10.1128/mSphere.00976-20 (PMC7771232; doi:10.1128/mSphere.00976-20)

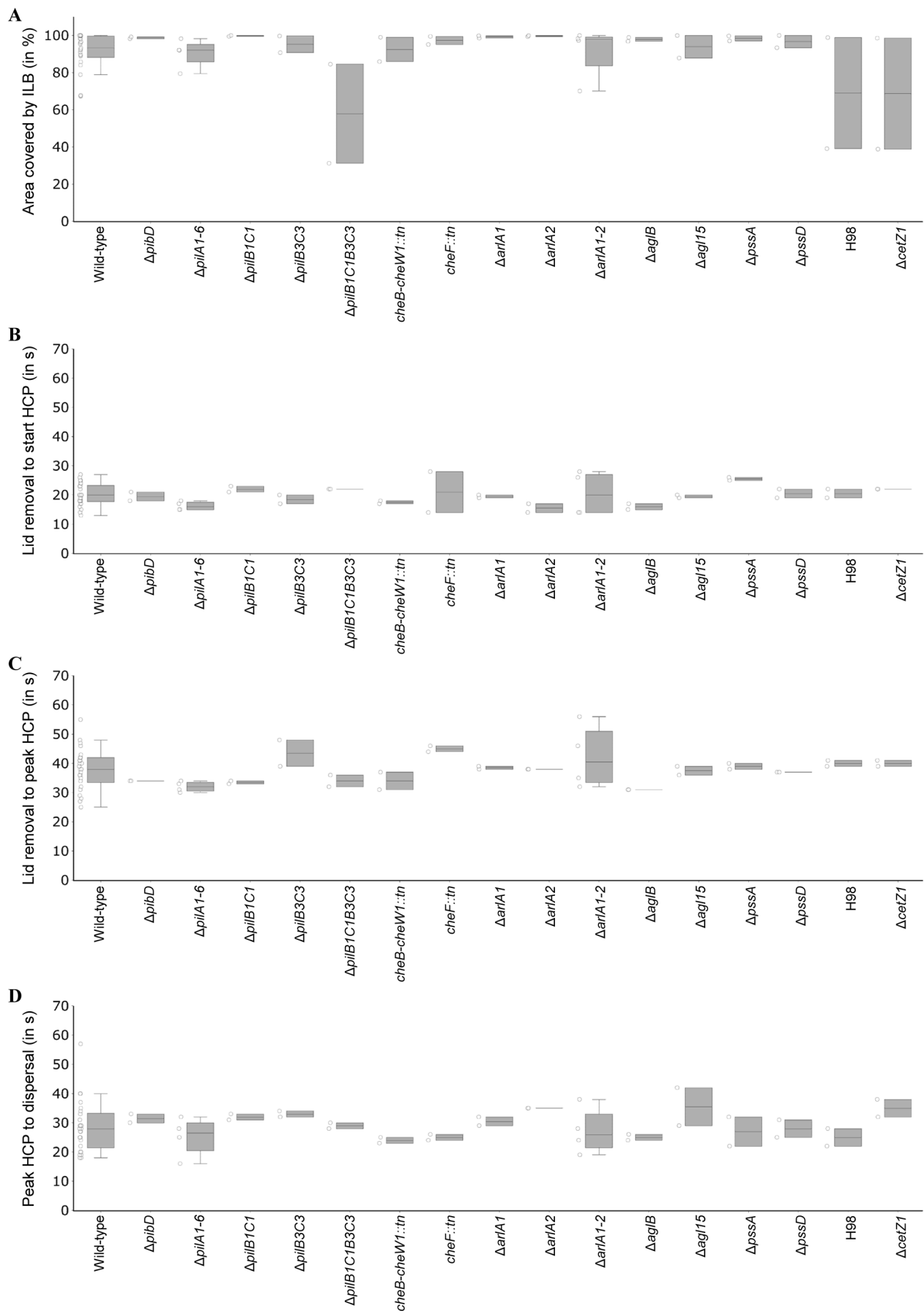

Supplement: FIG S1 [file mSphere.00976-20-sf001.pdf]

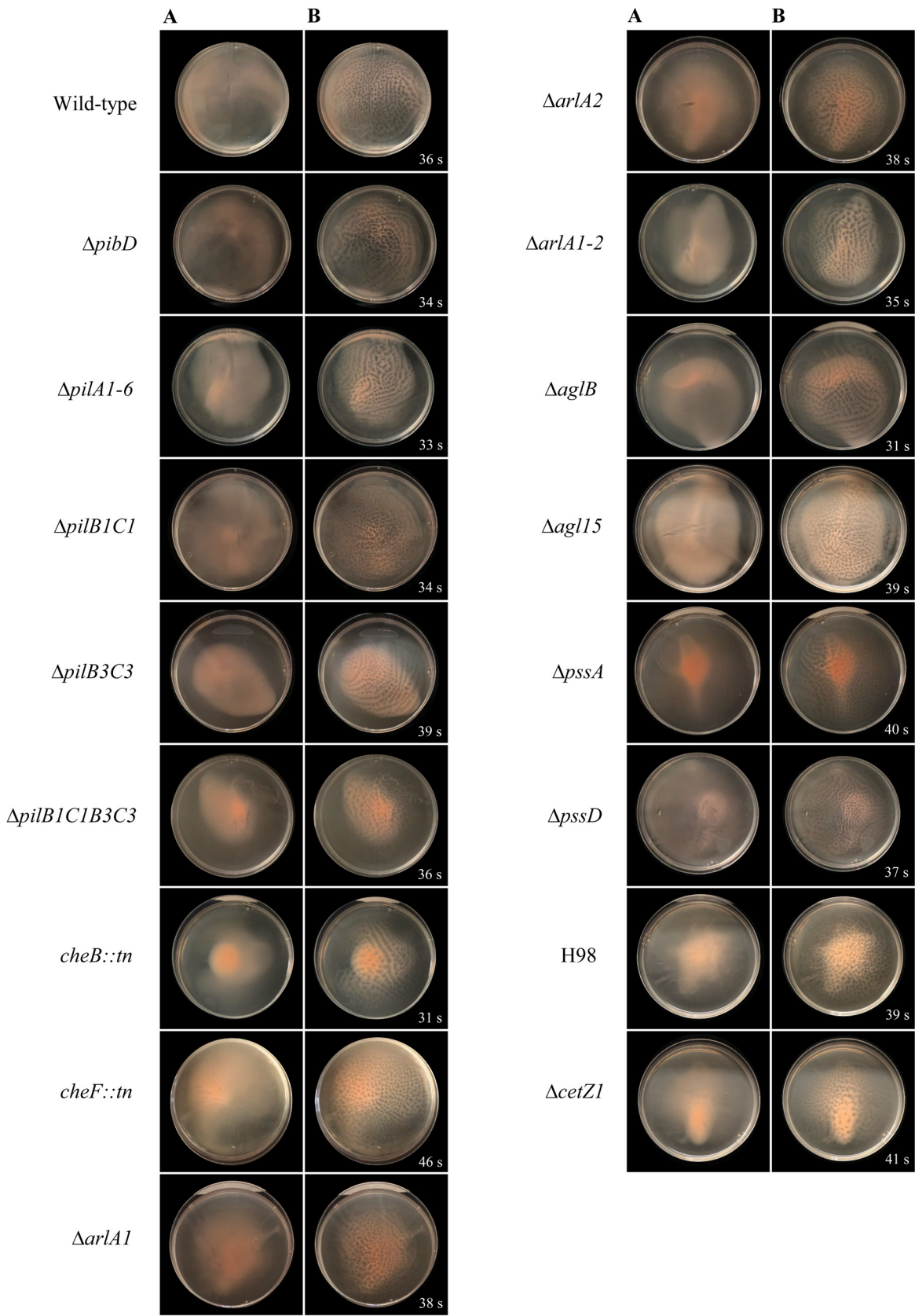

Supplement: FIG S2 [file mSphere.00976-20-sf002.pdf]

**A**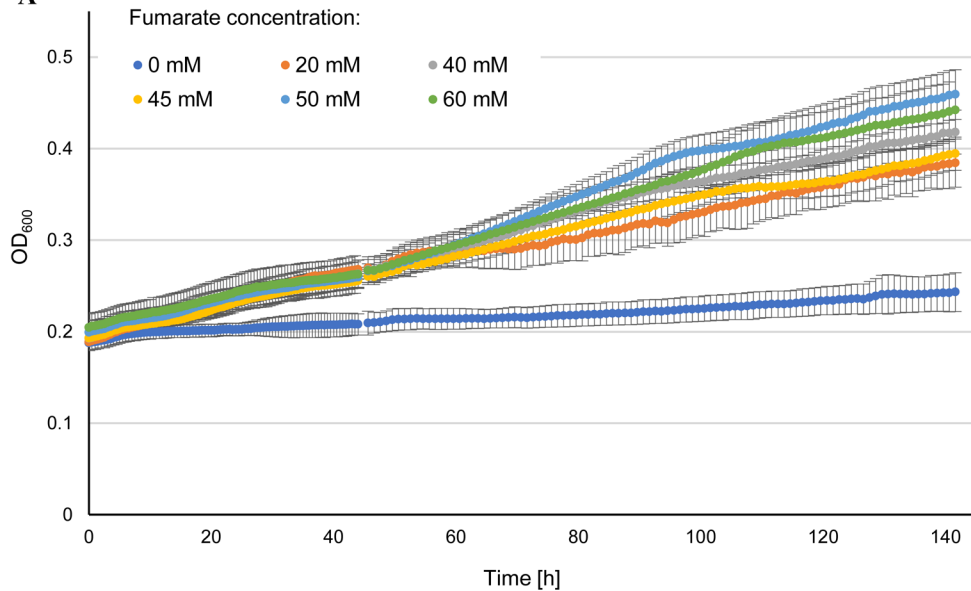**B**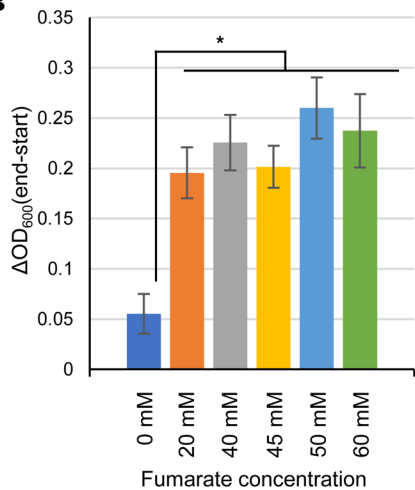**C**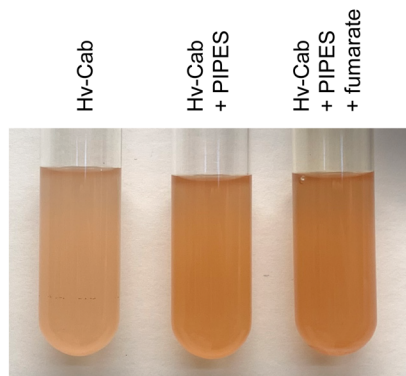

Supplement: FIG S3 [file mSphere.00976-20-sf003.pdf]

**A**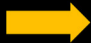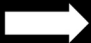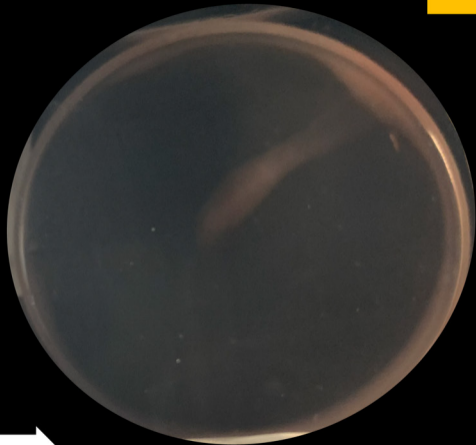**B**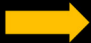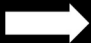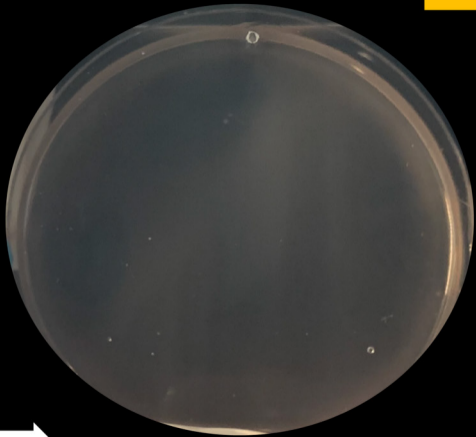

Supplement: FIG S4 [file mSphere.00976-20-sf004.pdf]

**A**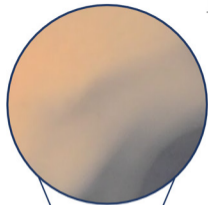**B**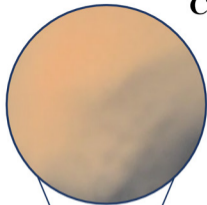**C**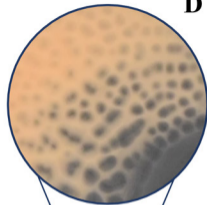**D**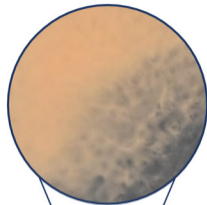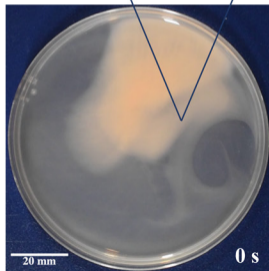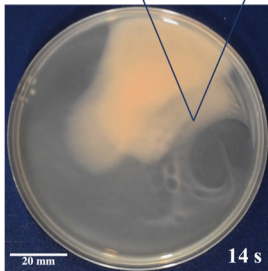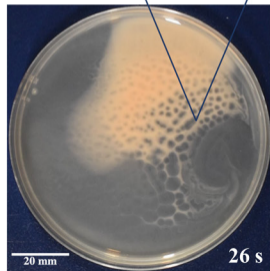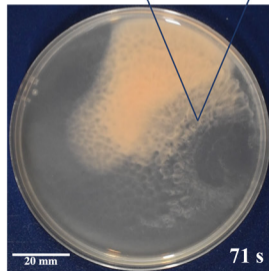

Supplement: FIG S5 [file mSphere.00976-20-sf005.pdf]
